# A Rare Case of Sclerosing Stromal Tumor of the Ovary Presenting in Pregnancy: A Diagnostic Dilemma on Presentation

**DOI:** 10.1155/2019/3927971

**Published:** 2019-12-30

**Authors:** Minh Nguyen, Namarig Soumit, Abdul Waheed, Jack Sees, Erum Azhar

**Affiliations:** ^1^WellSpan Good Samaritan Hospital Family Medicine Residency Program, Lebanon, PA, USA; ^2^WellSpan Pathology Associates, WellSpan Good Samaritan Hospital, Lebanon, PA, USA; ^3^Department of Obstetrics and Gynecology, Maimonides Medical Center, Brooklyn, NY, USA; ^4^Department of Public Health Sciences, Penn State Health Milton S Hershey Medical Center, Hershey, PA, USA

## Abstract

Sclerosing stromal tumor (SST) is a rare benign neoplasm of the ovary. There are only a few cases of sclerosing stromal tumor of the ovary during pregnancy that have been reported in the literature. The presenting symptoms are nonspecific, including pelvic pain or menstrual irregularities. We describe a case of a young 22-year-old pregnant woman who presented with pelvic pain in the second trimester. On imaging she was found to have a 12 cm left adnexal mass with solid features on MRI. The patient underwent exploratory laparotomy and removal of the mass that was attached to the left ovary via a stalk with preservation of the left ovary. The frozen section diagnosis was “sex cord stromal tumor, favor benign”. The final pathology confirmed the diagnosis of the sclerosing stromal tumor of the ovary where characteristic features of SST including a heterogenous, pseudolobular growth pattern with hypercellular and hypocellular areas were identified along with prominent luteinized stromal cells attributed to pregnancy. In this case report and review of literature, we emphasize consideration of this rare ovarian tumor in the differential diagnosis for a young pregnant woman who presents with pelvic pain.

## 1. Background

Sclerosing stromal tumor (SST) of the ovary is a benign neoplasm that was first reported in 1973 by Chalvardjan and Scully [1]. The occurrence is very rare, reported to account for about 6% of sex-cord stromal ovarian tumor subtypes [2, 3]. It usually occurs in women in the second or third decades of life, with 70% of the cases being reported to occur between 14 and 29 [4]. Sclerosing Stromal tumors are hormonally inactive [5]. However, rare hormonal activity has been reported in the literature [6] Ozdemir et al. reported a total of 208 cases of sclerosing stromal tumor from 2003 to 2014 with only 15 reported cases during pregnancy [7].

## 2. Case Presentation

A 22-year-old G1P0000 at 19 weeks and 5 days of gestation presented to the labor and delivery triage with severe left sided pelvic pain that started earlier that day. Pain was initially intermittent that became constant, severe, and stabbing in nature and lasted for 2 hours, thus prompting a visit to the labor and delivery floor. Patient reported nausea, vomiting, and nonbloody, loose bowel movements for 2 days prior to presentation as well. Patient reported her pain was worse with lying down and got better with leaning forward. She denied fever, sick contacts, trauma, chest pain, shortness of breath, vaginal bleeding, contractions, leakage of fluid, and decreased fetal movement. Prenatal labs were unremarkable.

The past medical history was remarkable for history of attention deficit disorder. Surgical history was significant for appendectomy performed in the current pregnancy at 7 weeks of gestation. The review of her chart revealed that she had an ultrasound at the time of appendectomy that showed the left ovary measuring 4.9 × 5.2 × 4.7 cm containing hemorrhagic corpus luteum cyst with normal right ovary. At the 12 weeks' nuchal translucency scan, the left ovary was measuring 6.3 × 5.4 × 5.1 cm.

Vitals in triage for this acute visit were in normal range. On physical exam, patient was in moderate discomfort with severe bilateral lower abdominal tenderness to palpation without rebound or guarding. No masses were felt due to gravid uterus. Genitourinary exam revealed closed cervix with left adnexal tenderness on pelvic examination. Fetal status was reassuring and patient had no contractions.

## 3. Investigations

**Figure 1:**
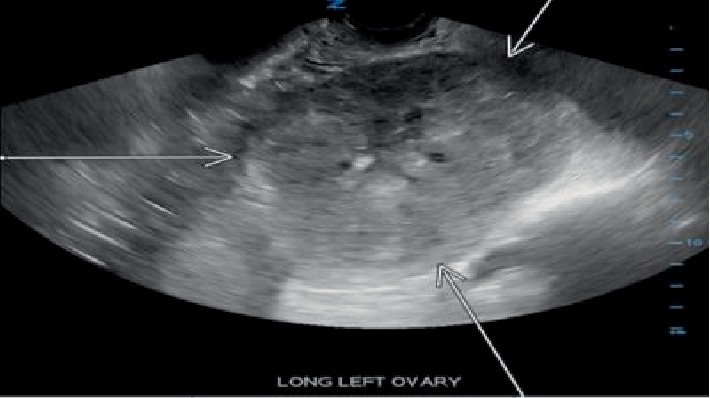
Ultrasound showed a solid hypervascular, mostly hypoechoic, partially isoechoic/partially hypoechoic left ovarian mass measuring 12.6 × 9.8 × 9.4 cm (outline by arrows).

Pelvic ultrasound showed a solid hypervascular, mostly hypoechoic, partially isoechoic/partially hypoechoic left ovarian mass measuring 12.6 × 9.8 × 9.4 cm, which had been persistently increasing in size over the course of the pregnancy (Figure 1). Peak arterial systolic velocity was 10 cm/sec. There was no complete or fixed ovarian torsion with presence of doppler color flow, but could not rule out intermittent torsion.

**Figure 2:**
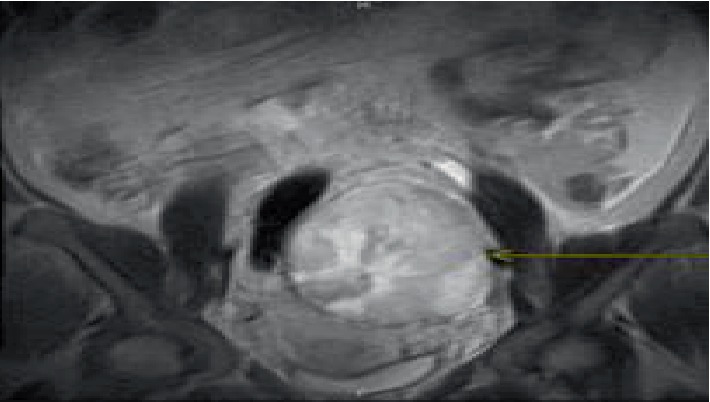
MRI pelvis without contrast coronal view revealed heterogeneous T2 hyperintense left ovarian mass measuring 12 × 9 × 10 cm (outlined by arrow).

MRI pelvis without contrast revealed heterogeneous T2 hyperintense and T1 isointense-to-hypointense left ovarian mass measuring 12 × 9× 10 cm; this mass was encapsulated with minimal cystic/fluid component centrally (Figure 2). There were no macroscopic fat or fluid-fluid levels to indicate dermoid or endometrioma, respectively. There was no classic cyst formation to suggest cystic neoplasm. There were no large intralesional flow voids to suggest enlarged/hyperemic neoplastic vasculature. The right ovary was not well visualized.

## 4. Differential Diagnosis

Differentials included gynecological and nongynecological causes of acute pelvic pain during second trimester. This included, but were not limited to, ovarian torsion, placental abruption, ruptured hemorrhagic cyst, round ligament pain, degenerating fibroids, and ovarian tumors both benign and malignant including sex cord stromal, epithelial, and germ cell tumors.

## 5. Treatment

Patient was admitted and underwent exploratory laparotomy. There was a large amount of clear peritoneal fluid noted in the abdominal cavity. A 15 cm left adnexal mass adherent to posterior cul-de-sac was seen. The ovary was removed from the posterior cul-de-sac using blunt dissection. The mass appeared to be attached to the left ovary by a small stalk which was cauterized and cut with LigaSure device. The left ovary appeared to be normal and was left behind intact. The right ovary appeared normal on inspection.

**Figure 3:**
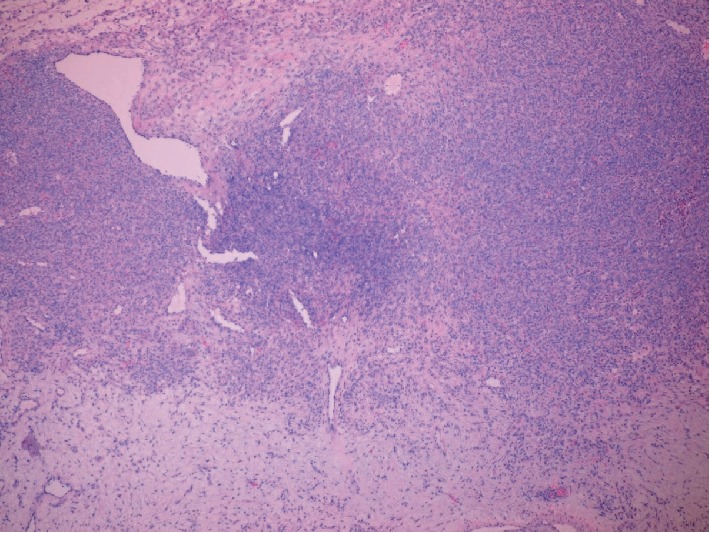
(40x, H&E) Pseudolobular pattern with variable cellularity and edema.

**Figure 4:**
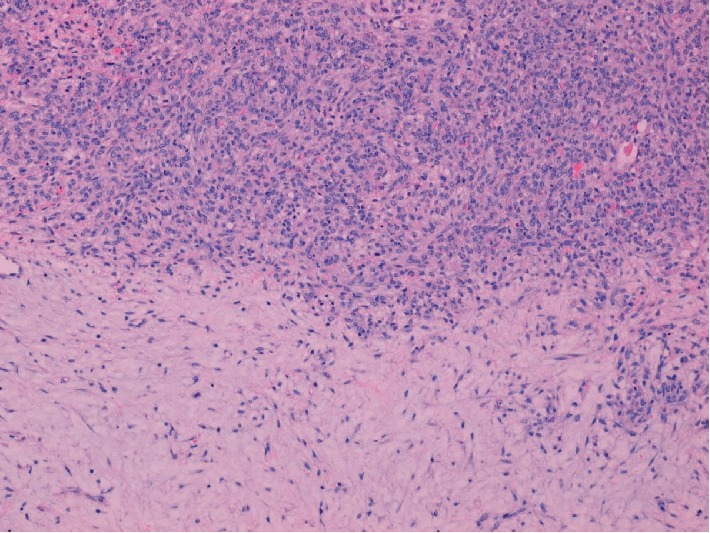
(100x, H&E) Hypercellular and hypocellular areas with edema.

**Figure 5:**
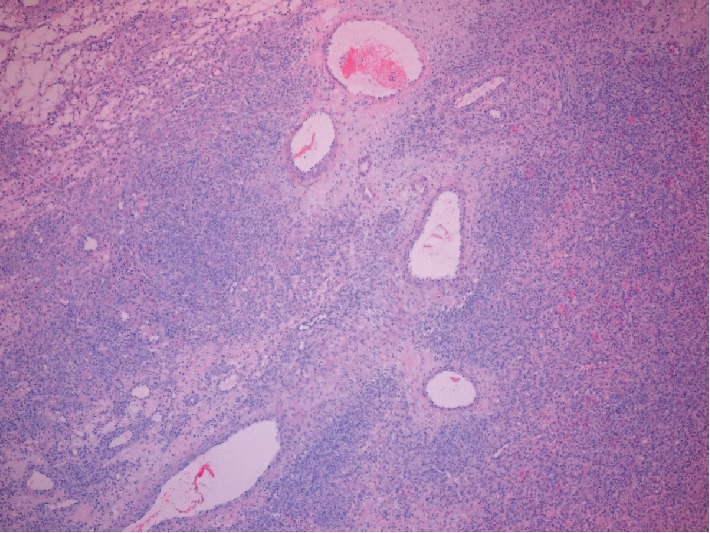
(40x, H&E) Prominent vascularity.

**Figure 6:**
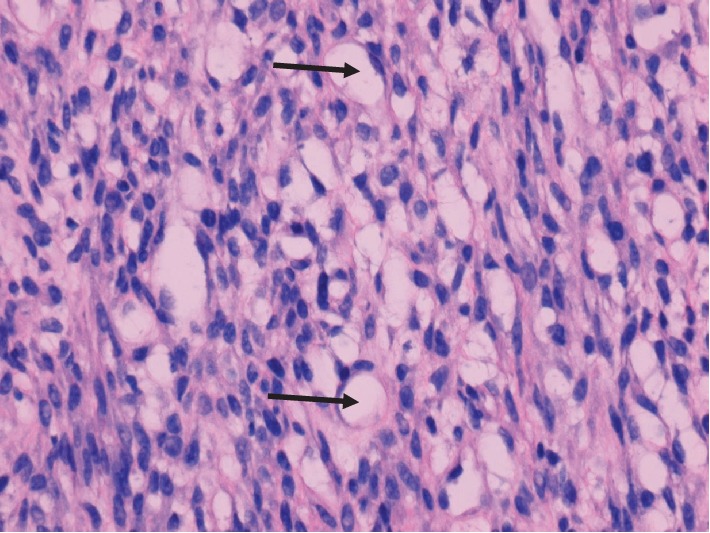
(400x, H&E) Round to oval clear cells with signet ring appearance admixed with spindled cells.

**Figure 7:**
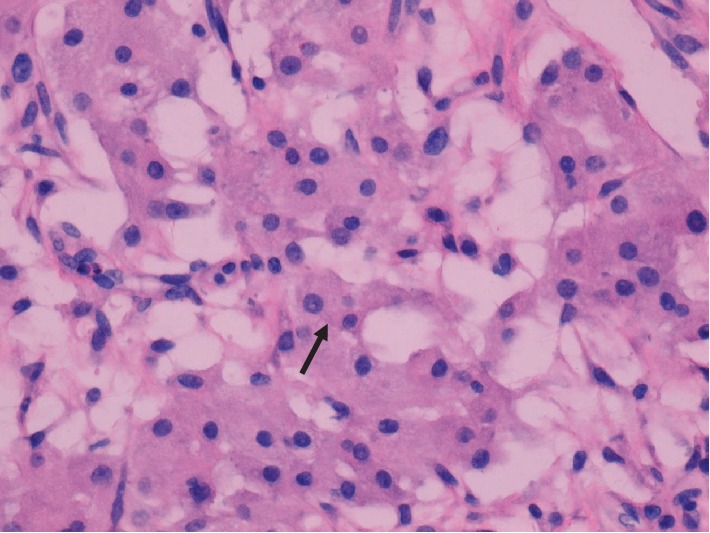
(400x, H&E) Plump, eosinophilic luteinized cells (black arrow).

**Figure 8:**
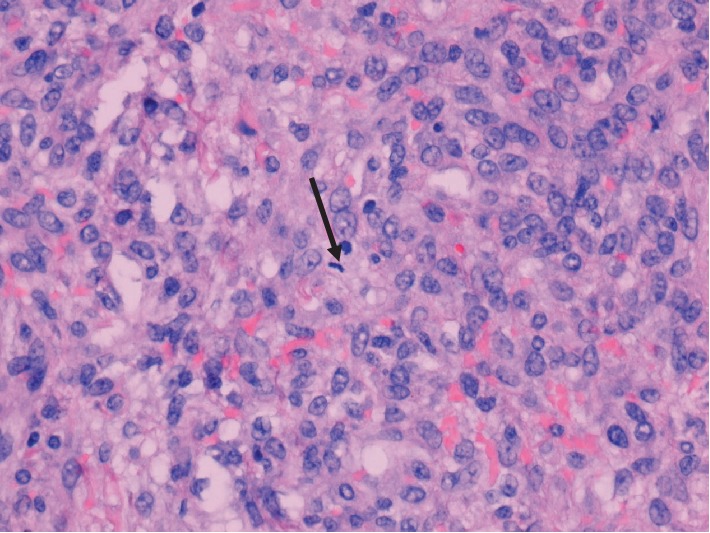
Microscopic section on (400x, H&E) reveals stromal cells with rare mitotic figure (black arrow).

Pathologic examination revealed a 436 gram, solid, gray-tan mass with a focal infarct. Microscopic features included a pseudolobular pattern with alternating hypercellular and hypocellular areas and edema (Figures 3 and 4), fibrosis, and a prominent vascular pattern (Figure 5). The cells include gland, spindle-shaped stromal cells, and prominent luteinized cells, including some with a signet ring appearance (Figure 6) as well as scattered collections of luteinized stromal cells with plump, eosinophilic cytoplasm (Figure 7). Occasional mitotic figures were identified, numbering up to 4 mitoses per 10 high power fields in the more cellular areas (Figure 8). There was no significant cytologic atypia.

## 6. Outcome and Follow-Up

Post operatively, patient did well. She continued with the pregnancy with no further complication, and had a normal spontaneous vaginal delivery of a healthy baby with no postpartum issues.

## 7. Discussion

Sex cord stromal tumors (SST) of the ovaries are rare ovarian neoplasms accounting for approximately 5–8% of ovarian tumors. They originate from sex cords and the ovarian stroma or mesenchyme. This group of ovarian tumors includes granulosa cell tumors, fibroma, thecoma, steroid cell tumors, Sertoli–Leydig cells tumors, sclerosing stromal tumors, and other morphologically indifferent cells [8–11]. Sclerosing stromal tumors comprise of approximately 6% of sex cord stromal tumors, and were first described in 1973 by Chalvardijan and Scully [1, 9, 10, 12]. These tumors are benign and rare, and their unique features can be distinguished from other stromal tumors by pathology and radiology [2].

Sclerosing stromal tumors are rarely seen in pregnancy making this a unique case [10]. Ozdemir et al. reported a total of 208 cases of sclerosing stromal tumor from 2003 to 2014 with only 15 reported cases during pregnancy [7]. Unlike other stromal ovarian tumors, sclerosing stromal tumors frequently occur in the second and third decades of life [2, 13]. Other stromal tumors of the ovaries occur in the fifth or sixth decade [13]. Common clinical presentations of sclerosing stromal tumors include menstrual irregularities, pelvic pain, and symptoms associated with pelvic mass that are nonspecific, and in majority of cases they occur unilaterally [2, 13].

Sclerosing stromal tumors of the ovaries are generally hormonally inactive; however, in the literature there have been some cases of tumors that are hormonally active. These active hormonal tumors may have estrogenic and rarely androgenic effect [2,6]. The active tumor produces dehydroepiandrosterone which causes menstrual irregularity, amenorrhea, infertility, precocious puberty, and virilization [7]. In these cases, the estrogenic and androgenic effects resolve after surgery [7]. In the literature, virilization has been noted in 9 cases to date, three of which were in pregnant women [7]. There also have been reported cases of ascites and elevated CA 125 [4,7]. In our patient, hormone levels were not obtained due to acute presentation.

On ultrasound sclerosing stromal tumor appears as a solid hypervascular mass with hypoechoic area in the center (Figure 1). Several vessels that are predominantly peripheral are seen on doppler [2]. This predominance is noted to be directed toward the center appearing as a “spoke-wheel” [2]. On MRI sclerosing stromal tumors appear as heterogeneous on T2 weighted images with hyperintense area. On T1 weighted images it appears as isointense-to-hypointense mass.

Sclerosing stromal tumors demonstrate a characteristic constellation of histologic findings including a pseudolobular growth pattern with variably cellular areas, edema, prominent vascularity that has been described as hemangiopericytoma-like in appearance, and stromal cells admixed with luteinized cells. Luteinized stromal cells are recognized by their oval and round shape and originally eosinophilic to clear, lipid containing cytoplasm. In pregnancy, Luteinized stromal cells can become quite pronounced and create diagnostic confusion, in some cases suggesting consideration of a signet ring carcinoma or Krukenberg tumor.

In most instances, clinical context and recognition of characteristic histologic features of SST will avoid misdiagnosis. In difficult cases, special stains and immunohistochemistry stains can be employed to allow destruction. Mucicarmine and immunohistochemistry epithelial markers, such as Pankeratin will decorate metastatic signet ring carcinoma cells, while luteinized stromal cells will be negative.

Sclerosing stromal tumors are rare, benign ovarian neoplasm occurring in young females. Clinical, radiological, and morphological findings are needed to make a diagnosis and to differentiate them from other types of sex cord stromal neoplasms [8]. Histological findings confirm the diagnosis. This sex cord ovarian neoplasm should be considered in young females who present with related symptoms and have a unilateral, solid/cystic, complex ovarian mass on radiological imaging [8]. Sclerosing stromal tumors have a good prognosis and can be treated by enucleation or unilateral oophorectomy [5]. In this case, the patient underwent ovarian conservation with a favorable clinical outcome. We emphasize fertility sparing surgery for these young women, as the very few studies in literature that are reported show absence of SST recurrence [14–16].

## 8. Conclusion

Sclerosing stromal tumor (SST) is a benign neoplasm of the ovary and can present with nonspecific symptoms such as pelvic pain in pregnancy. It is importance to consider all possibilities related or unrelated to pregnancy while evaluating a patient's complaint during pregnancy. The morphologic histopathology can pose diagnostic dilemma in pregnancy if there is an abundance of luteinized cells, raising a differential diagnosis that may include malignancy. Intraoperative frozen section can be used to confirm the benign nature of the neoplasm and allow a conservative, ovary sparing surgical approach.

## References

[1] Chalvardjian A., Scully R. E. (1973). Sclerosing stromal tumors of the ovary. *Cancer*.

[2] Palmeiro M. M., Cunha T. M., Loureiro A. L., Esteves G. (2016). A rare benign ovarian tumour. *BMJ Case Report*.

[3] Stylianidou A., Varras M., Akrivis C., Fylaktidou A., Stefana-ki S., Antoniou N. (2001). Sclerosing stromal tumor of the ovary: a case report and review of the literature. *European Journal Gynaecological Oncology*.

[4] Kim J. Y., Jung K. J., Chung D. S., Kim O. D., Lee J. H., Youn S. K. (2003). Sclerosing stromal tumor of the ovary: MR-pathologic correlation in three cases. *Korean Journal of Radiology*.

[5] Atram M., Anshu S. S., Gangane N. (2014). Sclerosing stromal tumor of the ovary. *Obstetrics & Gynecology Science*.

[6] Lam R. M., Geittmann P. (1988). Sclerosing stromal tumor of the ovary: a light, electron microscopic and enzyme histo-chemical study. *International Journal of Gynecological Pathology*.

[7] Ozdemir O., Sarı M. E., Sen E., Kurt A., Ileri A. B., Atalay C. R. (2014). Sclerosing stromal tumour of the ovary: a case report and the review of literature. *Nigerian Medical Journal*.

[8] Bairwa S., Satarkar R. N., Kalhan S., Garg S., Sangwaiya A., Singh P. (2017). Sclerosing stromal tumor: a rare ovarian neoplasm. *Iranian Journal of Pathology*.

[9] Atram M., Anshu Sharma S., Gangane N. (2014). Sclerosing stromal tumor of the ovary. *Obstetrics & Gynecology Science*.

[10] Iravanloo G., Nozarian Z., Sarrafpour B., Scully R. E. (2008). Sclerosing stromal tumor of the ovary. *Archives of Iranian Medicine*.

[11] Berek J., Hacker N. (2005). *Practical Gynecologic Oncology*.

[12] Young R. H. (2018). Ovarian sex cord stromal tumor. *Archives of Pathology & Laboratory Medicine*.

[13] Khanna M., Khanna A., Manjari M. (2012). Sclerosing stromal tumor of ovary: a case report. *Case Reports in Pathology*.

[14] Haroon S., Zia A., Idrees R., Memon A., Fatima S., Kayani N. (2013). Clinicopathological spectrum of ovarian sex cord-stromal tumors; 20 years’ retrospective study in a developing country. *Journal of Ovarian Research*.

[15] Grechi G., Clemente N., Tozzi A., Ciavattini A. (2015). Laparoscopic treatment of sclerosing stromal tumor of the ovary in a woman with Gorlin-Goltz syndrome: a case report and review of the literature. *Journal of Minimally Invasive Gynecology*.

[16] Abd El hafez A. (2014). Sclerosing stromal tumor of the ovary: a rare entity with distinctive features. *Case Reports in Clinical Pathology*.

[17] Bennett J. A., Oliva E., Young R. H. (2015). Sclerosing stromal tumors with prominent luteinization during pregnancy. *International Journal of Gynecological Pathology*.

